# Supplementary material for: Changes in PHQ-9 depression scores in acute stroke patients shortly after returning home
Source: PLoS One. 2021 Nov 11;16(11):e0259806. doi: 10.1371/journal.pone.0259806 (PMC8584969; doi:10.1371/journal.pone.0259806)
Supplement: S1 File — (DOCX) [file pone.0259806.s001.docx]

SUPPORTING INFORMATION

**Changes in PHQ-9 depression scores in acute stroke patients shortly after**

**returning home**

Brent Strong, Michele C. Fritz, Liming Dong, Lynda D. Lisabeth, Mathew J. Reeves

**Table of Contents**

1. Supplemental methods
   - 1. Full list of candidate predictors.
     2. Details of IPW sensitivity analysis.
2. Supplemental results
   - 1. Table A. STROBE checklist.
     2. Table B. Patient demographics and clinical characteristics by PHQ-9 completion at 7 days.
     3. Table C. Patient demographic and clinical characteristics by PHQ-9 completion at 90 days.
     4. Table D. Multivariable linear regression analysis of PHQ-9 score at 7 days. Comparison of results with PHQ-9 scores collected by proxy included and not included.
     5. Table E. Multivariable linear regression analysis of PHQ-9 score at 7 days. Comparison of results with IPW weighting applied and not applied.
     6. Table F. Multivariable linear regression analysis of change in PHQ-9 score from 7 to 90 days (interaction of predictors with time). Comparison of results with PHQ-9 scores collected by proxy included and not included.
     7. Table G. Multivariable linear regression analysis of change in PHQ-9 score from 7 to 90 days (interaction of predictors with time). Comparison of results with IPW weighting applied and not applied.
3. Supplemental references

**Supplemental methods**

Candidate predictors

The following variables were identified as candidate predictors of depressive symptoms: age, race, sex, education, marital status, living alone, history of depression, stroke type, stroke severity, modified Rankin Scale score at discharge, length of stay in the hospital, discharge destination, length of stay in rehabilitation, randomization arm, enrolled caregiver, and caregiver relationship. Education was ultimately not assessed due to a high frequency of missing data. Caregiver relationship was also not evaluated because it was used to impute missing values for marital status.

Inverse probability weighting (IPW) analysis

We conducted the IPW analysis to account for missing outcome data using methods similar to those outlined by Weuve et al.[[1](#_ENREF_1)] and Seaman and White.[[2](#_ENREF_2)] Weighting was done at the observation level. First, a set of plausible predictors of both missingness and PHQ-9 score were identified. These included age (specified as a categorical variable), sex, race, stroke severity, disability at baseline, and history of depression. We also included history of prior stroke, length of stay in the hospital, and six-item cognitive screen score at baseline because they strongly predicted missingness at either 7 or 90 days (P<0.05). A square root transformation was applied to both length of stay in the hospital and six-item cognitive screen score. We first used these covariates to fit a logistic regression model on the outcome of remaining in the study at 7 days. The Hosmer-Lemeshow test did not indicate poor fit (P=0.97). From this logistic regression model, we generated predicted probabilities of remaining in the study at 7 days which were trimmed at the 1^st^ and 99^th^ percentiles. Weights for observations at 7 days were then taken to be the inverse of the predicted probabilities such that patients with a lower predicted probability of remaining in the study had higher weights.

In constructing the weights for 90-day observations, we adopted the approach of Weuve et al. and considered any patient with missing data at 7 days to also have missing data at 90 days.[[1](#_ENREF_1)] This was done so that the missingness pattern was monotone and resulted in 21 patients being assigned a value of “missing” for their 90-day PHQ-9 score even though it was collected. We fit a logistic regression model on the outcome of remaining in the study at 90 days, conditional on remaining in the study at 7 days. Because the number of patients that dropped out between the 7- and 90-day interviews was small, only a subset of covariates from the 7-day model were used. We selected these covariates for inclusion in the model if they were associated with missingness at 90-days (conditional on remaining in the study at 7 days) at P<0.20. The final model consisted of age, history of prior stroke, six-item cognitive screen score at baseline (square-root transformed), and PHQ-9 score at 7 days. The Hosmer-Lemeshow test did not indicate poor fit (P=0.58), and we again trimmed the predicted probabilities at the 1^st^ and 99^th^ percentile. The weights for observations at 90 days were taken to be the product of the weight for the corresponding observation at 7-days (i.e., the observation from the same patient) and the inverse of the predicted probability of remaining in the study at 90 days (conditional on remaining in the study at 7 days). The final, multivariable generalized estimating equation was then generated with the weights applied. We used an independence working correlation structure instead of an exchangeable working correlation structure (which we used in our unweighted models) as recommended by Tchetgen Tchetgen et al.[[3](#_ENREF_3)] The statistical code to implement our IPW analysis is available at the Open Science Framework (<https://osf.io/4hzdf/>).

**Supplemental results**

Table A. STROBE checklist for cohort studies

|  | Item No | Recommendation | Page |
| --- | --- | --- | --- |
| **Title and abstract** | 1 | (*a*) Indicate the study’s design with a commonly used term in the title or the abstract | 2 |
|  |  | (*b*) Provide in the abstract an informative and balanced summary of what was done and what was found | 2 |
| Introduction | | |  |
| Background/rationale | 2 | Explain the scientific background and rationale for the investigation being reported | 3 |
| Objectives | 3 | State specific objectives, including any prespecified hypotheses | 3-4 |
| Methods | | |  |
| Study design | 4 | Present key elements of study design early in the paper | 4 |
| Setting | 5 | Describe the setting, locations, and relevant dates, including periods of recruitment, exposure, follow-up, and data collection | 4-5 |
| Participants | 6 | (*a*) Give the eligibility criteria, and the sources and methods of selection of participants. Describe methods of follow-up | 4-5 |
|  |  | (*b*) For matched studies, give matching criteria and number of exposed and unexposed | N/A |
| Variables | 7 | Clearly define all outcomes, exposures, predictors, potential confounders, and effect modifiers. Give diagnostic criteria, if applicable | 5-6, S1 Supporting Information |
| Data sources/ measurement | 8* | For each variable of interest, give sources of data and details of methods of assessment (measurement). Describe comparability of assessment methods if there is more than one group | 5-6 |
| Bias | 9 | Describe any efforts to address potential sources of bias | 7 |
| Study size | 10 | Explain how the study size was arrived at | 4 (references 11 & 12 for more details) |
| Quantitative variables | 11 | Explain how quantitative variables were handled in the analyses. If applicable, describe which groupings were chosen and why | 5-7 |
| Statistical methods | 12 | (*a*) Describe all statistical methods, including those used to control for confounding | 6-7 |
|  |  | (*b*) Describe any methods used to examine subgroups and interactions | 7 |
|  |  | (*c*) Explain how missing data were addressed | 7 |
|  |  | (*d*) If applicable, explain how loss to follow-up was addressed | 7 |
|  |  | (*e*) Describe any sensitivity analyses | 7 |
| Results | | |  |
| Participants | 13* | (a) Report numbers of individuals at each stage of study—eg numbers potentially eligible, examined for eligibility, confirmed eligible, included in the study, completing follow-up, and analysed | 7 (reference 11 for more details) |
|  |  | (b) Give reasons for non-participation at each stage | 7 (reference 11 for more details) |
|  |  | (c) Consider use of a flow diagram | N/A |
| Descriptive data | 14* | (a) Give characteristics of study participants (eg demographic, clinical, social) and information on exposures and potential confounders | Table 1 |
|  |  | (b) Indicate number of participants with missing data for each variable of interest | 5, 7 |
|  |  | (c) Summarise follow-up time (eg, average and total amount) | 5 |
| Outcome data | 15* | Report numbers of outcome events or summary measures over time | Table 1 |
| Main results | 16 | (*a*) Give unadjusted estimates and, if applicable, confounder-adjusted estimates and their precision (eg, 95% confidence interval). Make clear which confounders were adjusted for and why they were included | Tables 2 & 3 |
|  |  | (*b*) Report category boundaries when continuous variables were categorized | Tables 2 & 3 |
|  |  | (*c*) If relevant, consider translating estimates of relative risk into absolute risk for a meaningful time period | N/A |
| Other analyses | 17 | Report other analyses done—eg analyses of subgroups and interactions, and sensitivity analyses | 13-14, 16 |
| Discussion | | |  |
| Key results | 18 | Summarise key results with reference to study objectives | 16 |
| Limitations | 19 | Discuss limitations of the study, taking into account sources of potential bias or imprecision. Discuss both direction and magnitude of any potential bias | 17, 19 |
| Interpretation | 20 | Give a cautious overall interpretation of results considering objectives, limitations, multiplicity of analyses, results from similar studies, and other relevant evidence | 16-18 |
| Generalisability | 21 | Discuss the generalisability (external validity) of the study results | 17-18, 19 |
| Other information | | |  |
| Funding | 22 | Give the source of funding and the role of the funders for the present study and, if applicable, for the original study on which the present article is based | online |

*Give information separately for exposed and unexposed groups.

**Note:** An Explanation and Elaboration article discusses each checklist item and gives methodological background and published examples of transparent reporting. The STROBE checklist is best used in conjunction with this article (freely available on the Web sites of PLoS Medicine at http://www.plosmedicine.org/, Annals of Internal Medicine at http://www.annals.org/, and Epidemiology at http://www.epidem.com/). Information on the STROBE Initiative is available at http://www.strobe-statement.org.

Table B. Patient demographics and clinical characteristics by PHQ-9 completion status at 7 days.

| **Variable*** | **PHQ-9 Score Collected** | **PHQ-9 Score Missing** | **P-value**† |
| --- | --- | --- | --- |
| Number of patients | 193 | 72 | N/A |
| Site  Sparrow Hospital  St. Joseph Mercy Ann Arbor  University of Michigan Hospital | 117 (61%)  51 (26%)  25 (13%) | 36 (50%)  22 (31%)  14 (19%) | 0.23 |
| Age  18-59  60-69  70-79  >=80 | 50 (26%)  62 (32%)  52 (27%)  29 (15%) | 24 (33%)  18 (25%)  15 (21%)  15 (21%) | 0.29 |
| Sex  Male  Female | 98 (51%)  95 (49%) | 36 (50%)  36 (50%) | 1.00 |
| Race  White  Non-white | 161 (83%)  32 (17%) | 49 (68%)  23 (32%) | **0.01** |
| Living Alone Prior to Stroke (n=263)  No  Yes | 143 (75%)  48 (25%) | 54 (75%)  18 (25%) | 1.00 |
| Caregiver consented  No  Yes | 65 (34%)  128 (66%) | 31 (43%)  41 (57%) | 0.20 |
| Stroke type  Ischemic  Hemorrhagic | 169 (88%)  24 (12%) | 58 (81%)  14 (19%) | 0.17 |
| Stroke severity (NIHSS & GCS) ‡  Mild  Moderate  Severe | 141 (73%)  41 (21%)  11 (6%) | 49 (68%)  15 (21%)  8 (11%) | 0.31 |
| Discharge mRS  Mild (<2)  Moderate/Severe (3+) | 131 (68%)  62 (32%) | 47 (65%)  25 (35%) | 0.77 |
| Discharge Destination  Home or community  Inpatient rehabilitation  Sub-acute rehabilitation | 88 (46%)  87 (45%)  18 (9%) | 30 (42%)  36 (50%)  6 (8%) | 0.82 |
| History of Depression  No  Yes | 169 (88%)  24 (12%) | 61 (85%)  11 (15%) | 0.54 |
| Randomization Arm§  Usual Care  SWCM  SWCM+ | 60 (31%)  66 (34%)  67 (35%) | 27 (38%)  22 (31%)  23 (32%) | 0.63 |
| Acute LOS  Median (IQR) | 4 (2, 5) | 5 (3,8) | **0.005** |
| Rehab LOS  Median (IQR) | 6 (0, 15) | 8 (0, 16.5) | 0.41 |
| Prior Stroke  No  Yes | 168 (87%)  25 (13%) | 56 (78%)  16 (22%) | 0.08 |
| Diabetes Mellitus  No  Yes | 123 (64%)  70 (36%) | 45 (63%)  27 (38%) | 0.89 |
| Hypertension  No  Yes | 43 (22%)  150 (78%) | 17 (24%)  55 (76%) | 0.87 |
| 6-item Cognitive Screen Score  Median (IQR) \| \| | 6 (5, 6) | 5 (5, 6) | 0.10 |

*We were not able to assess the differences in the number of married patients because marital status was ascertained during the 7-day interview when PHQ-9 scores were collected.

†P-values were calculated with a Fisher’s exact test for categorical variables and a Wilcoxon two-sample test for continuous variables. Significant p-values are bolded.

‡ Stroke severity was categorized as mild (National Institutes of Health Stroke Scale [NIHSS] 1-5, or Glasgow Coma Scale [GCS] 13-15), moderate (NIHSS 6-13, or GCS 5-12), and severe (NIHSS 14-42, or GCS 3-4).

§ SWCM indicates the social worker case management arm, and SWCM+ indicates the social worker case management plus website arm.

| | For 11 patients missing a baseline cognitive screen score, a score of 4 was imputed because all 11 patients required proxy consent. The highest cognitive screen score for which a proxy consent was sought was 4.

Table C. Patient demographics and clinical characteristics by PHQ-9 completion status at 90 days.

| **Variable*** | **PHQ-9 Score Collected** | **PHQ-9 Score Missing** | **P-value**† |
| --- | --- | --- | --- |
| Number of patients | 185 | 80 | n/a |
| Site  Sparrow  St. Joe  U-M | 112 (61%)  51 (28%)  22 (12%) | 41 (51%)  22 (28%)  17 (21%) | 0.13 |
| Age  18-59  60-69  70-79  >=80 | 53 (29%)  58 (31%)  48 (26%)  26 (14%) | 21 (26%)  22 (28%)  19 (24%)  18 (23%) | 0.42 |
| Sex  Male  Female | 93 (50%)  92 (50%) | 41 (51%)  39 (49%) | 0.89 |
| Race  White  Non-white | 153 (83%)  32 (17%) | 57 (71%)  23 (29%) | **0.05** |
| Living Alone Prior to Stroke (n=263)  No  Yes | 135 (74%)  48 (26%) | 62 (78%)  18 (23%) | 0.64 |
| Caregiver consented  No  Yes | 65 (35%)  120 (65%) | 31 (39%)  49 (61%) | 0.58 |
| Stroke type  Ischemic  Hemorrhagic | 160 (86%)  25 (14%) | 67 (84%)  13 (16%) | 0.57 |
| Stroke severity (NIHSS & GCS) ‡  Mild  Moderate  Severe | 136 (74%)  36 (19%)  13 (7%) | 54 (68%)  20 (25%)  6 (8%) | 0.55 |
| Discharge mRS  Mild (<2)  Moderate/Severe (3+) | 127 (69%)  58 (31%) | 51 (64%)  29 (36%) | 0.48 |
| Discharge Destination  Home or community  Inpatient rehabilitation  Sub-acute rehabilitation | 87 (47%)  83 (45%)  15 (8%) | 31 (39%)  40 (50%)  9 (11%) | 0.39 |
| History of Depression  No  Yes | 163 (88%)  22 (12%) | 67 (84%)  13 (16%) | 0.33 |
| Randomization Arm§  Usual Care  SWCM  SWCM+ | 58 (31%)  64 (35%)  63 (34%) | 29 (36%)  24 (30%)  27 (34%) | 0.69 |
| Acute LOS  Median (IQR) | 4 (2, 6) | 5 (3, 8) | **0.02** |
| Rehab LOS  Median (IQR) | 5 (0, 16) | 7.5 (0, 15.5) | 0.30 |
| Prior Stroke  No  Yes | 163 (88%)  22 (12%) | 61 (76%)  19 (24%) | **0.03** |
| Diabetes Mellitus  No  Yes | 122 (66%)  63 (34%) | 46 (58%)  34 (43%) | 0.21 |
| Hypertension  No  Yes | 46 (25%)  139 (75%) | 14 (18%)  66 (83%) | 0.20 |
| 6-item Cognitive Screen Score  Median (IQR) \| \| | 6 (5, 6) | 5 (5, 6) | **0.02** |

*We were not able to assess the differences in the number of married patients because marital status was ascertained during the 7-day interview when PHQ-9 scores were collected.

† P-values were calculated with a Fisher’s exact test for categorical variables and a Wilcoxon two-sample test for continuous variables. Significant p-values are bolded.

‡Stroke severity was categorized as mild (National Institutes of Health Stroke Scale [NIHSS] 1-5, or Glasgow Coma Scale [GCS] 13-15), moderate (NIHSS 6-13, or GCS 5-12), and severe (NIHSS 14-42, or GCS 3-4).

§ SWCM indicates the social worker case management arm, and SWCM+ indicates the social worker case management plus website arm.

| | For 11 patients missing a baseline cognitive screen score, a score of 4 was imputed because all 11 patients required proxy consent. The highest cognitive screen score for which a proxy consent was sought was 4.

Table D. Multivariable linear regression analysis of PHQ-9 score at 7 days. Comparison of results with PHQ-9 scores collected by proxy included and excluded.

| **Predictor** | **Adjusted β (95% CI), Proxy Observations Included (n=193 patients at 7 days)*** | **Adjusted β (95% CI), Proxy Observations Excluded (n=179 patients at 7 days)*** |
| --- | --- | --- |
| *Age* | | |
| 18-59 | 0 (ref) | 0 (ref) |
| 60-69 | -0.7 (-2.6, 1.2) | -0.8 (-2.7, 1.1) |
| 70-79 | -2.4 (-4.2, -0.6) | -2.2 (-4.1, -0.3) |
| >80 | -2.4 (-4.7, -0.1) | -3.2 (-5.4, -1.0) |
| *Sex* | | |
| Male | 0 (ref) | 0 (ref) |
| Female | 1.5 (0.1, 3.0) | 1.9 (0.4, 3.4) |
| *Race* | | |
| White | 0 (ref) | 0 (ref) |
| Non-white | 1.7 (0.0, 3.5) | 1.9 (0.1, 3.7) |
| *Marital Status* | | |
| Married | 0 (ref) | 0 (ref) |
| Not Married | -1.7 (-3.1, -0.3) | -2.2 (-3.6, -0.8) |
| *Discharge Destination* | | |
| Home | 0 (ref) | 0 (ref) |
| Acute Rehab | 0.7 (-0.7, 2.2) | 0.7 (-0.8, 2.1) |
| Sub-acute Rehab | 2.7 (0.1, 5.4) | 2.5 (0.0, 5.0) |
| *Randomization Arm*† | | |
| Usual Care | 0 (ref) | 0 (ref) |
| SWCM | -0.1 (-1.8, 1.6) | -0.3 (-1.9, 1.4) |
| SWCM+ | 0.4 (-1.4, 2.1) | 0.2 (-1.5, 1.9) |
| *Stroke Severity* | | |
| Mild | 0 (ref) | 0 (ref) |
| Moderate | -2.4 (-3.8, -1.0) | -1.9 (-3.4, -0.5) |
| High | -0.7 (-2.9, 1.4) | 0.2 (-2.4, 2.9) |

* A positive value indicates elevated depressive symptoms at baseline (7 days after discharge). Predictors are adjusted for all other predictors included in the table.

† SWCM indicates the social worker case management arm, and SWCM+ indicates the social worker case management plus website arm.

Table E. Multivariable linear regression analysis of PHQ-9 score at 7 days. Comparison of results with and without IPW weighting.

| **Predictor** | **Adjusted β (95% CI), IPW Weighting Not Applied*** | **Adjusted β (95% CI), IPW Weighting Applied*** |
| --- | --- | --- |
| *Age* | | |
| 18-59 | 0 (ref) | 0 (ref) |
| 60-69 | -0.7 (-2.6, 1.2) | -0.9 (-2.8, 0.9) |
| 70-79 | -2.4 (-4.2, -0.6) | -2.5 (-4.4, -0.6) |
| >80 | -2.4 (-4.7, -0.1) | -2.4 (-4.8, 0.0) |
| *Sex* | | |
| Male | 0 (ref) | 0 (ref) |
| Female | 1.5 (0.1, 3.0) | 1.3 (-0.2, 2.8) |
| *Race* | | |
| White | 0 (ref) | 0 (ref) |
| Non-white | 1.7 (0.0, 3.5) | 2.0 (0.2, 3.8) |
| *Marital Status* | | |
| Married | 0 (ref) | 0 (ref) |
| Not Married | -1.7 (-3.1, -0.3) | -1.7 (-3.2, -0.2) |
| *Discharge Destination* | | |
| Home | 0 (ref) | 0 (ref) |
| Acute Rehab | 0.7 (-0.7, 2.2) | 0.6 (-0.9, 2.0) |
| Sub-acute Rehab | 2.7 (0.1, 5.4) | 2.8 (-0.2, 5.8) |
| *Randomization Arm*† | | |
| Usual Care | 0 (ref) | 0 (ref) |
| SWCM | -0.1 (-1.8, 1.6) | -0.3 (-2.1, 1.5) |
| SWCM+ | 0.4 (-1.4, 2.1) | 0.4 (-1.4, 2.2) |
| *Stroke Severity* | | |
| Mild | 0 (ref) | 0 (ref) |
| Moderate | -2.4 (-3.8, -1.0) | -2.4 (-3.8, -1.0) |
| High | -0.7 (-2.9, 1.4) | -0.2 (-2.3, 1.9) |

* A positive value indicates elevated depressive symptoms at baseline (7 days after discharge). Predictors are adjusted for all other predictors included in the table.

† SWCM indicates the social worker case management arm, and SWCM+ indicates the social worker case management plus website arm.

Table F. Multivariable linear regression analysis of change in PHQ-9 score from 7 to 90 days (2-way interaction of predictors with time). Comparison of results with PHQ-9 scores collected by proxy included and not included.

| **Predictor** | **Adjusted β (95% CI), Proxy Observations Included (n=378 observations)*** | **Adjusted β (95% CI), Proxy Observations Not Included (n=353 observations)*** |
| --- | --- | --- |
| *Age* | | |
| 18-59 | 0 (ref) | 0 (ref) |
| 60-69 | 0.0 (2.0, 2.0) | 0.8 (-1.0, 2.6) |
| 70-79 | -0.9 (-2.7, 1.0) | -0.4 (-2.1, 1.3) |
| ≥80 | 0.0 (-2.1, 2.2) | 1.3 (-0.7, 3.3) |
| *Sex* | | |
| Male | 0 (ref) | 0 (ref) |
| Female | -0.5 (-1.9, 0.9) | -0.7 (-2.1, 0.6) |
| *Race* | | |
| White | 0 (ref) | 0 (ref) |
| Non-white | -1.3 (-3.1, 0.5) | -1.2 (-3.0, 0.6) |
| *Marital Status* | | |
| Married | 0 (ref) | 0 (ref) |
| Unmarried | 3.2 (1.9, 4.6) | 3.9 (2.5, 5.2) |
| *Discharge Destination* | | |
| Home | 0 (ref) | 0 (ref) |
| Acute Rehab | 0.7 (-0.7, 2.1) | 0.2 (-1.1, 1.4) |
| Sub-acute Rehab | -1.0 (-3.7, 1.8) | -1.0 (-3.7, 1.7) |
| *Randomization Arm*† | | |
| Usual Care | 0 (ref) | 0 (ref) |
| SWCM | -0.5 (-2.1, 1.0) | -0.5 (-1.9, 1.0) |
| SWCM+ | -0.2 (-1.9, 1.5) | -0.6 (-2.1, 1.0) |
| *Stroke Severity* | | |
| Mild | 0 (ref) | 0 (ref) |
| Moderate | 3.3 (1.7, 5.0) | 3.6 (2.0, 5.3) |
| High | 3.2 (0.5, 5.9) | 1.9 (-1.1, 4.8) |

* A positive value indicates an increase in depressive symptoms over time relative to the reference group. Predictors are adjusted for all other predictors included in the table.

† SWCM indicates the social worker case management arm, and SWCM+ indicates the social worker case management plus website arm.

Table G. Multivariable linear regression analysis of change in PHQ-9 score from 7 to 90 days (interaction of predictors with time). Comparison of results with IPW weighting not applied and applied.

| **Predictor** | **Adjusted β (95% CI), IPW Weighting Not Applied*** | **Adjusted β (95% CI), IPW Weighting Applied*** |
| --- | --- | --- |
| *Age* | | |
| 18-59 | 0 (ref) | 0 (ref) |
| 60-69 | 0.0 (2.0, 2.0) | -1.0 (-3.2, 1.3) |
| 70-79 | -0.9 (-2.7, 1.0) | -1.8 (-4.1, 0.4) |
| ≥80 | 0.0 (-2.1, 2.2) | -1.2 (-4.5, 2.0) |
| *Sex* | | |
| Male | 0 (ref) | 0 (ref) |
| Female | -0.5 (-1.9, 0.9) | -0.6 (-2.3, 1.1) |
| *Race* | | |
| White | 0 (ref) | 0 (ref) |
| Non-white | -1.3 (-3.1, 0.5) | -1.0 (-3.3, 1.3) |
| *Marital Status* | | |
| Married | 0 (ref) | 0 (ref) |
| Unmarried | 3.2 (1.9, 4.6) | 3.4 (1.6, 5.2) |
| *Discharge Destination* | | |
| Home | 0 (ref) | 0 (ref) |
| Acute Rehab | 0.7 (-0.7, 2.1) | 0.8 (-0.9, 2.5) |
| Sub-acute Rehab | -1.0 (-3.7, 1.8) | -1.1 (-4.6, 2.4) |
| *Randomization Arm*† | | |
| Usual Care | 0 (ref) | 0 (ref) |
| SWCM | -0.5 (-2.1, 1.0) | -0.8 (-2.8, 1.2) |
| SWCM+ | -0.2 (-1.9, 1.5) | 0.3 (-2.0, 2.6) |
| *Stroke Severity* | | |
| Mild | 0 (ref) | 0 (ref) |
| Moderate | 3.3 (1.7, 5.0) | 3.0 (1.1, 5.0) |
| High | 3.2 (0.5, 5.9) | 4.2 (1.2, 7.2) |

* A positive value indicates an increase in depressive symptoms over time relative to the reference group. Predictors are adjusted for all other predictors included in the table.

† SWCM indicates the social worker case management arm, and SWCM+ indicates the social worker case management plus website arm.

**Supplemental references**

1. Weuve J, Tchetgen Tchetgen EJ, Glymour MM, Beck TL, Aggarwal NT, et al. Accounting for bias due to selective attrition: the example of smoking and cognitive decline. Epidemiology. 2012;23: 119-128.

2. Seaman SR, White IR. Review of inverse probability weighting for dealing with missing data. Stat Methods Med Res. 2013;22: 278-295.

3. Tchetgen Tchetgen EJ, Glymour MM, Weuve J, Robins J. Specifying the correlation structure in inverse-probability-weighting estimation for repeated measures. Epidemiology. 2012;23: 644-646.
