# Supplementary material for: Changes in PHQ-9 depression scores in acute stroke patients shortly after returning home
Source: PLoS One. 2021 Nov 11;16(11):e0259806. doi: 10.1371/journal.pone.0259806 (PMC8584969; doi:10.1371/journal.pone.0259806)
Supplement: Attachment — Submitted filename: Response to Reviewers.docx [file pone.0259806.s002.docx]

September 1, 2021

Seana Gall, PhD

Academic Editor, *PLOS ONE*

Re: Changes in PHQ-9 Depression Scores in Acute Stroke Patients Shortly After

Returning Home

Dear Dr. Gall:

We would like to extend our gratitude for the review and comments we received in your letter dated August 9 on the above referenced paper. We are resubmitting the attached manuscript, which has been formatted to meet *PLOS ONE*’s requirements. The full name of the IRB that approved the study has been added to the ethics statement since this was missing previously. The updated ethics statements can be found in the Methods section, page 5, lines 83-85. Furthermore, in our first submission, we did not provide a data availability statement written in full sentences. Our updated data availability statement is the following: “All data files and analytic code are available at the Open Science Framework (<https://osf.io/4hzdf/)>.” Below is our response to the reviewer’s comments. All page and line numbers refer to the clean version of the manuscript.

Reviewer 1

Thank you for the opportunity to review this manuscript. I found the manuscript interesting, clearly presented and well explained. The objective of the study is to identify the factors associated with the severity of depressive symptoms at 7 days and 90 days post-discharge. The authors performed a secondary analysis of patients enrolled in the MISTT trial.

Response: Thank you for these positive comments.

Question for the authors: What is your reason for choosing to measure depressive symptoms at 7 days and 90 days post-discharge? Is there any evidence in the literature that shows stroke survivors develop depressive symptoms as early as 7 days? or within 7-90 days post-discharge? I think it will be good to have this justification.

Response: Thank you for raising an excellent point. Fortunately, robust data on the time course of depression after acute stroke is provided by a 2014 meta-analysis of 61 observational studies that reported the frequency of depression relative to stroke onset (see reference 7 [Hackett and Pickles, 2014] in our paper). At 0-1 month after stroke, the pooled prevalence of depression was 28%, and at 2-5 months, it was 36%. According to data from the National Health Interview Survey (https://www.cdc.gov/nchs/data/databriefs/db379-H.pdf), the prevalence of moderate or severe depression (corresponding to a PHQ-8 score of ≥10, the most commonly used cutoff for post-stroke depression) in the general US adult population is less than 8% for every age group. The much higher prevalence of depression among stroke patients at 0 to 1 month after onset compared to the general population strongly suggests that post-stroke depression can be observed within a short time after stroke. These observations justify our examination of factors that are associated with both baseline depressive symptoms as well as change over time (since the meta-analysis also indicates that the prevalence of post stroke depression increases over time). We have revised the Introduction section to emphasize these important details.

New text (Introduction, page 3, lines 47-49): *The prevalence of post-stroke depression stroke has been well studied in various settings; a 2014 meta-analysis found that the pooled prevalence of depression was 28% at 0 to 1 month after stroke onset and 36% at 2 to 5 months after stroke onset (Hackett et al., 2014).*

**Reviewer 2**

The authors have presented an interesting study regarding the changes in PHQ-9 in acute stroke patients shortly after returning home and reported that being unmarried and having higher stroke severity was associated with fewer depressive symptoms at baseline, but both factors were associated with worsening depressive symptoms over time. I have just a few queries.

Response: Thank you for both the positive comments and helpful suggestions.

Could the authors please explain the reason for line in Results section (quoted below) where moderate and mild stroke are compared but not severe stroke?
“After adjustment, older age, being unmarried, and having moderate stroke severity (versus mild) were significantly associated with lower baseline PHQ-9 scores,…”

Response: In this sentence we were highlighting which factors were significantly associated with baseline PHQ-9 score. Stroke severity was specified as a three-level ordinal variable, but only the comparison between moderate and mild strokes (the referent category) was statistically significant. The comparison between high severity strokes and mild strokes is provided in Table 2, but we did not highlight it in this sentence because it was not statistically significant.

Relevant portion of Table 2:

It is mentioned that female sex is an independent predictor of the outcomes. Did authors examine any interaction of sex with severity of stroke that could add to the findings?

Response: Thank you for raising this important point. In our original analysis, we examined the interaction of the two strongest predictors (marital status and severity) with key demographic variables (age, sex, and race). Both two-way interactions (for PHQ-9 score at baseline) and three-way interactions including time (for change in PHQ-9 score) were tested. In the case of sex and stroke severity, both the interaction terms were not significant (P=0.24 and P=0.28). However, the two-way interaction between age and marital status was significant (P=0.045). We have added language to the Methods section that reports our examination of interactions and explains that we did not include any significant interactions in the final model because they were exploratory in nature and the sample size prevented us from drawing reliable inferences about their direction and magnitude. Model fit was also better without the interactions. However, we do now describe the one significant interaction test result (age by marital status) in the results section.

New text (Methods, page 7, lines 137-141): *We also tested interactions between important predictor variables (namely, stroke severity and marital status) and age, sex, and race. Although we describe the general conclusions of any significant interactions, we did not incorporate these interactions into the final model because they were exploratory, and the sample size was too small to make reliable inferences about their direction and magnitude.*

New text (Methods, page 13, lines 208-212): *The only significant interaction at baseline was between age and marital status. The observation that unmarried patients had lower 7-day PHQ-9 scores than married patients was limited to the 60 to 69 and 70 to 79 age groups. Conversely, unmarried patients had higher PHQ-9 scores than married patients in the age group over 80.*

What was the proportion of missing data overall? Could that information be added in the discussion?

Response: We report the proportion of missing data at the beginning of the Results section and acknowledge the high rate of missing data as a limitation in the Discussion. The relevant text is provided below.

Relevant text (Results, page 7, lines 147-149): *Of 265 patients enrolled in MISTT, PHQ-9 data was available for 193 (73%) at 7 days, and 185 (70%) at 90 days. A total of 214 patients (81%) completed at least one PHQ-9 survey, and 164 (62%) completed both for a total of 378 observations.*

Relevant text (Discussion, page 19, line 308-310): *Although the rate of missing data was relatively high, when missing participants were accounted for using IPW, changes in model results were minimal.*

The checklist in the supplementary data is missing page numbers after third page and needs to be completed.

Response: Thank you for pointing this out. For whatever reason, the original Word document for the supplementary data has these page numbers, but they seem to be missing when the Word file is downloaded from *PLOS ONE.* We’ve taken steps to ensure this doesn’t happen again.

Additional revisions

We made two more important changes to the manuscript:

1. We improved our use of inverse probability weighting (IPW) to account for missing data by refining our choice of covariates to include in the relevant statistical models, modifying how the weights were calculated, and changing the working correlation structure in the relevant generalized estimating equations. This was done after consulting pertinent references (references 1, 2, and 3 in the S1 Supporting Information). Extensive details on our updated methods are provided in the S1 Supporting Information. The results are virtually unchanged from those we reported for the IPW analysis in our original submission.

2. We now discuss selection bias as a possible explanation for the unexpected findings in relation to the association of marital status and severity with baseline PHQ-9 scores. The relevant text can be found in the Discussion (page 17, lines 269-273). This was added because we believe that selection bias is a plausible reason for our findings along with framing effects and response shift, which we discussed in the original version of the manuscript.

New text (Discussion, page 17, lines 269-273): *The first is selection bias, which can occur when an analysis is conditioned on a variable affected by both the exposure (or cause of the exposure) and outcome (or cause of the outcome) (Hernan et al., 2004). Participation in the MISTT study required informed consent, which is plausibly affected by a patient’s stroke severity, marital status, and depressive symptoms in the hospital. Thus, selection bias could have been introduced at the point of initial enrollment.*

Finally, the following changes to the reference section have been made. All reference number refer to the track changes version:

1. Use of reference 8 (Hackett and Pickles, 2014) in the introduction instead of another meta-analysis (reference 7 [Mitchell et al., 2017]) which we cited in our original submission when we discussed the prevalence of post-stroke depression. This substitution was done because reference 8 (Hackett and Pickles, 2014) provides pooled estimates of post-stroke depression prevalence at various time points after onset, which helped us to address the comment from reviewer 1.

2. Addition of reference 21 (Seaman and White, 2013) to the sentence in the methods discussing our use of inverse probability weighting (IPW). We used this article extensively to revise our IPW analysis.

3. Addition of reference 25 (Hernan et al., 2004) to support our discussion of selection bias.

In conclusion, we have responded to the reviewers’ comments to the best of our ability and believe that the paper has been significantly improved. All authors have read and approved this version of the manuscript. Please don’t hesitate to reach out with any questions or concerns.

Yours sincerely,

Brent Strong and Mathew Reeves

Department of Epidemiology and Biostatistics,

Michigan State University
